# Improvement in Quality-of-Life-Related Outcomes Following Treatment with IncobotulinumtoxinA in Adults with Limb Spasticity: A Pooled Analysis

**DOI:** 10.3390/toxins16010019

**Published:** 2023-12-29

**Authors:** Franco Molteni, Jörg Wissel, Klemens Fheodoroff, Michael C. Munin, Atul T. Patel, Michael Althaus, Georg Comes, Andrzej Dekundy, Irena Pulte, Astrid Scheschonka, Matteo Vacchelli, Andrea Santamato

**Affiliations:** 1 Department of Rehabilitation, Valduce Villa Beretta Hospital, 23845 Costa Masnaga, Italy; 2 Department of Neurorehabilitation and Physical Therapy, Vivantes Hospital Spandau, 13585 Berlin, Germany; 3 Gailtal-Klinik, A-9620 Hermagor, Austria; klemens.fheodoroff@gailtal-klinik.at; 4 Department of Physical Medicine and Rehabilitation, University of Pittsburgh School of Medicine, Pittsburgh, PA 15213, USA; 5 Kansas City Bone and Joint Clinic, Overland Park, KS 66211, USA; 6 Merz Therapeutics GmbH, 60318 Frankfurt am Main, Germany; michael.althaus@merz.de (M.A.); georg.comes@merz.de (G.C.);; 7 Unit of Spasticity and Movement Disorders, Division of Physical Medicine and Rehabilitation, University Hospital of Foggia, 71100 Foggia, Italy

**Keywords:** upper limb spasticity, botulinum toxin type A, incobotulinumtoxinA, limb position, dressing, hygiene, pooled analysis, quality-of-life-related outcomes

## Abstract

A strong correlation has been reported between patient-reported quality of life (QoL) and the investigator-rated Disability Assessment Scale (DAS) in patients with spasticity. The current analysis evaluates the effect of incobotulinumtoxinA on QoL-related outcomes (limb position abnormality, as well as dressing- and hygiene-related disability, measured with the DAS) in adults with upper limb spasticity, using pooled data from six studies. Separate analyses for each DAS domain were performed using data from patients with disabilities for that domain (DAS score ≥1). Results showed that a significantly greater proportion of incobotulinumtoxinA-treated compared with placebo-treated patients achieved a ≥1-point reduction from baseline in each of the DAS domains (improvement) 4 weeks after the first injection. The benefits of incobotulinumtoxinA were observed regardless of the baseline severity of DAS impairment and of the time elapsed since stroke. The effects of incobotulinumtoxinA 4 weeks after injection were maintained or enhanced over multiple injection cycles for all three DAS domains, supporting the use of repeated injection cycles to provide sustained QoL benefit. IncobotulinumtoxinA represents an important treatment option to achieve better QoL-related outcomes for patients with upper limb spasticity, irrespective of the duration of their condition.

## 1. Introduction

Spasticity is a sign of many central nervous system conditions, including stroke, multiple sclerosis, cerebral palsy, spinal cord injury, and traumatic brain injury [1,2,3,4,5,6]. Spasticity is estimated to occur in 25% of patients after stroke, rising to 40% in patients with paresis [7]. Upper limb spasticity affects coordinated movement; limits activities such as grasp, release, and self-care activities; and predisposes to secondary complications, such as joint contractures and pain [8,9].

Botulinum toxin type A (BoNT-A) is recommended as first-line treatment for spasticity [10,11]. The safety and efficacy of incobotulinumtoxinA have been demonstrated in a number of studies in the treatment of limb spasticity and reduction of spasticity-associated pain [12,13,14,15,16,17,18]. Additional investigations revealed that incobotulinumtoxinA is also associated with quality-of-life (QoL) improvement in adults with spasticity [16,19].

The Disability Assessment Scale (DAS) evaluates upper limb functional impairments and activity limitations commonly seen in patients with poststroke spasticity in four domains: limb position abnormality, dressing- and hygiene-related disability, and pain [20]. Higher DAS scores indicate greater impairments in each of the four domains [20]. Although the DAS was developed to assess poststroke spasticity-related impairments and activity limitations, its use is not only limited to stroke-related problems [21,22,23].

As previously demonstrated, upper limb spasticity has a significant negative effect on health-related QoL (HRQoL) [24]. A spasticity-specific QoL scale (the Patient Reported Impact of Spasticity Measure; PRISM) has been used to demonstrate that spasticity in general can result in social avoidance or anxiety, and more severe spasticity in the arm may be associated with higher levels of perceived social embarrassment [25]. A strong correlation has been reported between patient-reported HRQoL and the investigator-rated DAS dressing- and hygiene-related disability and pain domains [26] when used to classify problems relevant to patients with upper limb poststroke spasticity, which include hygiene, dressing, and limb posture. These DAS domains are, therefore, important to patients. Poststroke spasticity and the related limitations to performing basic activities can also have a societal, emotional, and economic impact on caregivers [27,28]. Regarding the DAS, caregiver burden and the need for assistance are directly related to the level of disability in the dressing and hygiene domains [26].

The impact of incobotulinumtoxinA on upper limb spasticity-related limb position abnormality, dressing and hygiene has been investigated using the DAS in several studies as a primary [16,18] or secondary outcome [12,14,15,17]. Most of these studies reported DAS outcomes with respect to the principal therapeutic target (PTT), which was the DAS domain identified by the patients in conjunction with the investigators as the most relevant and important for potential improvement with BoNT-A. The PTT was required to have a score ≥2 at screening/baseline.

The aim of the current study was to assess the benefit of treatment with incobotulinumtoxinA in adults with upper limb spasticity by considering its effect on QoL-related outcomes (limb position abnormality, dressing- and hygiene-related disability, as measured using the DAS), using pooled data from six phase 2 or 3 clinical studies. The pain domain data were excluded from this analysis since the effects of incobotulinumtoxinA on spasticity-associated pain from these six studies have recently been presented elsewhere [29]. We hypothesized that QoL-related outcomes would improve after treatment with BoNT-A in a similar manner to spasticity-associated pain [29]. To include the largest possible sample size and to assess whether domains that were not identified as the PTT also benefitted from incobotulinumtoxinA treatment, the analyses were based on all evaluable patients with data for the individual DAS items rather than just the PTT data.

## 2. Results

### 2.1. Baseline Characteristics

A total of 937 patients participated in the six studies [12,14,15,17,18,30], of whom 716 received incobotulinumtoxinA and 221 received a placebo for their first cycle of treatment in the respective studies. Of the 937 patients at baseline, 918 (98.0%) had upper limb position abnormality at baseline (incobotulinumtoxinA, *n* = 699; placebo, *n* = 219), rated as mild (10.5%), moderate (51.0%), or severe (38.6%). A total of 907 (96.8%) patients had dressing disability (incobotulinumtoxinA, *n* = 690; placebo, *n* = 217), rated as mild (15.6%), moderate (56.0%), or severe (28.5%). Finally, 865 (92.3%) patients had hygiene-related (incobotulinumtoxinA, *n* = 655; placebo, *n* = 210), rated as mild (19.1%), moderate (53.4%), or severe (27.5%).

Baseline characteristics were numerically similar between incobotulinumtoxinA- and placebo-treated patients with limb position abnormality, dressing- and hygiene-related disability (score ≥1) at baseline (Table 1). More than 60% of each domain group was male, mean age was approximately 56 years, more than half were BoNT-naïve, and stroke was the most common cause of spasticity (in >90% of cases).

Of the 937 patients in total, the PTT was upper limb position abnormality for 431 (46.0%), dressing for 250 (26.7%), hygiene for 173 (18.5%), and pain for 45 (4.8%). The baseline characteristics of these 899 patients with PTT-related disability are summarized in Supplementary Table S1.

### 2.2. Response Rates at Week 4 Post First Injection

**Figure 1:**
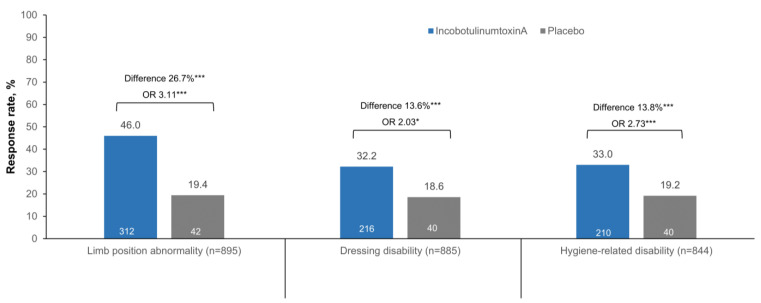
DAS domain response rates (≥1-point improvement in DAS score) at week 4 after the first injection cycle by treatment and domain. Chi-square test was used for OR and Wald test to determine between-treatment *p*-values. *, *p* < 0.01; ***, *p* < 0.0001. CI, confidence interval; DAS, Disability Assessment Scale; OR, odds ratio.

In patients with limb position abnormality at baseline, incobotulinumtoxinA treatment produced a higher response rate than placebo (46.0% vs. 19.4%, a difference (95% confidence interval (CI)) of 26.7 (20.2–33.1)%; *p* < 0.0001), with incobotulinumtoxinA-treated patients being at least three times more likely to achieve a response than placebo-treated patients after the first cycle (odds ratio (OR) 3.11, 95% CI 2.04–4.75; *p* < 0.0001) (Figure 1 and Figure S1). IncobotulinumtoxinA treatment also produced a higher response rate than placebo after the first cycle for patients with baseline dressing disability (32.2% vs. 18.6%, a difference (95% CI) of 13.6 (7.3–19.9)%; *p* < 0.0001) and hygiene-related disability (33% vs. 19.2%, a difference (95% CI) of 13.8 (7.3–20.3)%; *p* < 0.0001) (Figure 1). Additionally, incobotulinumtoxinA-treated patients were at least twice as likely to achieve a response as placebo-treated patients regarding baseline dressing disability (OR 2.03, 95% CI 1.32–3.12; *p* < 0.01) and hygiene-related disability (OR 2.73, 95% CI 1.77–4.21; *p* < 0.0001) after the first cycle (Figure 1).

**Figure 2:**
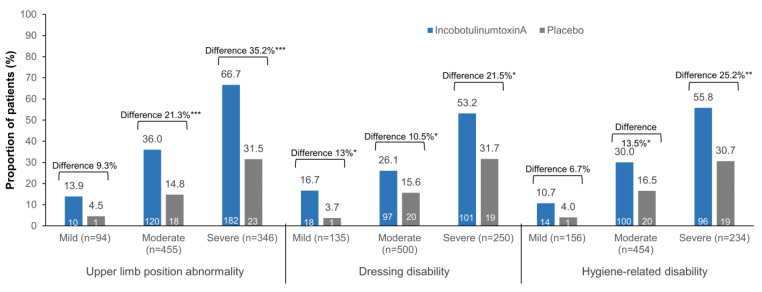
Differences in overall DAS domain response rates (≥1-point improvement in DAS score) at week 4 after the first injection cycle, by treatment and baseline severity of the respective DAS domain. Wald test was used to determine between-treatment *p*-values. *, *p* < 0.05; **, *p* < 0.001; ***, *p* < 0.0001. DAS, Disability Assessment Scale.

When analyzed by DAS domain severity at baseline, incobotulinumtoxinA-treated patients with moderate or severe abnormality showed a significantly higher response rate at week 4 than those treated with placebo (Figure 2). The difference in limb position abnormality response rate was 21.3% (95% CI 13.1–29.4; *p* < 0.0001) for moderate limb position abnormality at baseline and 35.2% (95% CI 23.1–47.2; *p* < 0.0001) for severe limb position abnormality at baseline. The difference in dressing disability response rate was 10.5% (95% CI 2.7–18.2; *p* = 0.0079) for moderate dressing disability at baseline and 21.5% (95% CI 7.7–35.2; *p* = 0.0022) for severe dressing disability at baseline. The difference in hygiene-related disability response rate was 13.5% (95% CI 5.3–21.7; *p* = 0.0013) for moderate hygiene-related disability at baseline and 25.2% (95% CI 11.5–38.8; *p* = 0.0003) for severe hygiene-related disability at baseline. Differences in response rates in patients with mild severity numerically favored incobotulinumtoxinA over placebo for all QoL-related outcomes investigated, reaching statistical significance for dressing disability (Figure 2).

In patients with stroke-related upper limb spasticity, time since stroke had little effect on the efficacy of incobotulinumtoxinA with respect to response rates at week 4 for each of the three DAS domains investigated (Table 2). Response rates were numerically similar across all time intervals assessed.

### 2.3. Response Rates Following Multiple Injection Cycles

**Figure 3:**
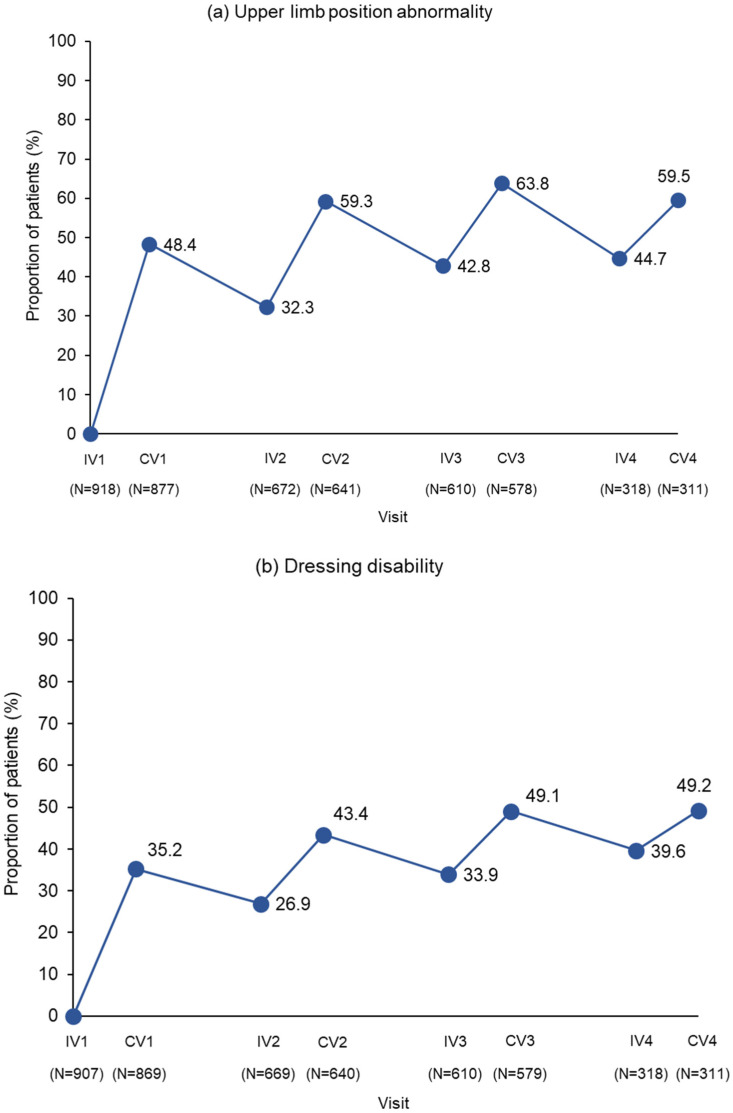

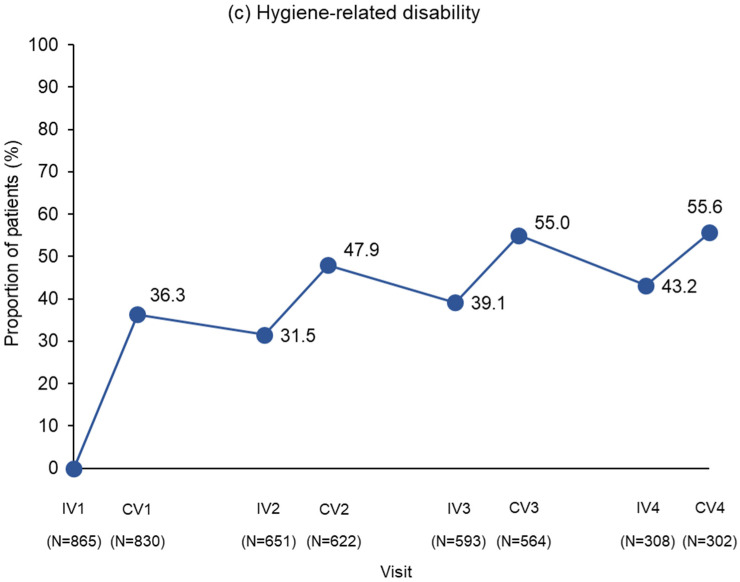
DAS domain response rates (≥1-point improvement in DAS score from baseline) at week 4 after repeated injection cycles. CV took place 4 weeks after the previous IV; time between injections was 12–14 weeks for the majority of patients. Only the first injection was placebo-controlled. As placebo-treated subjects received incobotulinumtoxinA in subsequent cycles, their responses to their first cycle of incobotulinumtoxinA (at study IV2 and CV2) are included in IV1 and CV1 and in the subsequent two incobotulinumtoxinA injection cycles. The N-value given below each visit is the number of patients with data at that visit. CV, control visit; DAS, Disability Assessment Scale; IV, injection visit.

Response rates for all three DAS domains investigated increased after each repeated incobotulinumtoxinA injection cycle, showing a cumulative positive effect on QoL-related outcomes (Figure 3). At each injection visit, many patients had a maintained response to incobotulinumtoxinA (>26%) and response rates at these visits increased with each subsequent injection cycle.

Four weeks after the fourth injection cycle, response rates in patients with limb position abnormality at baseline reached 59.5%. Response rates in patients with dressing disability reached 49.2%, and response rates in patients with hygiene-related disability reached 55.6%.

### 2.4. Response Rates for the PTT

In patients with a DAS disability domain as the PTT, a higher response rate was observed with incobotulinumtoxinA treatment than placebo 4 weeks after the first injection (52.0% vs. 25.8%, a difference (95% CI) of 26.2 (19.2–33.2)%; *p* < 0.0001). When analyzed by PTT severity at baseline (moderate or severe disability was a requirement), incobotulinumtoxinA-treated patients showed a significantly higher response rate at week 4 than those treated with placebo irrespective of severity: Response rates were 37.4% vs. 18.7% for those with moderate disability and 66.9% vs. 35.6% for those with severe disability (*p* < 0.0001 for both). Patients with 3–5, 6–10, and >10 years since stroke had numerically similar response rates with incobotulinumtoxinA compared to patients with 0–2 years since stroke (46.7%, 51.7%, and 64.5%, respectively, vs. 51.2%); all response rates were statistically higher with incobotulinumtoxinA than with placebo (*p* < 0.01 for all times since stroke).

Response rates for the PTT increased after repeated incobotulinumtoxinA injection cycles to 65.9% 4 weeks after the fourth injection, showing a cumulative positive effect. At each injection visit, many patients had a maintained response to incobotulinumtoxinA (36.9%–51.4%) and response rates at these visits increased with each subsequent injection cycle.

## 3. Discussion

This is one of the largest analyses to consider the effect of BoNT-A on individual DAS domains; a previous pooled analysis of data from the same studies confirmed the beneficial effect of incobotulinumtoxinA on the pain DAS domain [29]. Of the 937 patients included in these analyses, the vast majority had upper limb position abnormality (98.0%), dressing disability (96.8%) and hygiene-related disability (92.3%) at baseline, usually as a result of stroke-related spasticity. For many of the patients, these limitations were of sufficient severity to be considered as the PTT for treatment.

Results showed that a significantly greater proportion of incobotulinumtoxinA-treated compared with placebo-treated patients achieved a ≥1-point improvement in limb position abnormality, dressing- and hygiene-related disability DAS scores 4 weeks after the first injection. Compared with patients receiving a placebo, incobotulinumtoxinA-treated patients were at least three times more likely to achieve a reduction in DAS limb position abnormality score, almost three times more likely to achieve a reduction in DAS hygiene-related disability score, and at least twice as likely to achieve a reduction in DAS dressing disability score. Importantly, the benefits of incobotulinumtoxinA were observed regardless of the baseline severity of DAS impairment and, in those with stroke-related spasticity, of the time elapsed since stroke, although differences versus placebo were not always statistically significant in the subgroups. Specifically, patients with mild upper limb position abnormality or hygiene-related disability did not have statistically significantly higher response rates with incobotulinumtoxinA than with placebo. These nonsignificant findings may have been a result of the smaller numbers of patients with mild disability, the likelihood that individuals with mild disability have residual control of voluntary movements, which allows improvements in task performance in the placebo groups, or the fact that the opportunity for large improvements in those with only mild disability is reduced. Nonsignificant differences from placebo seen in time-since-stroke subgroups generally occurred with no discernible pattern, and again, patient numbers were reduced as time since stroke increased.

Time since stroke (<36 months vs. ≥36 months) was a significant predictor of active motor function in a study of onabotulinumtoxinA in upper limb spasticity, with a shorter time since stroke predicting a better functional response to treatment [31]. Furthermore, in a study of lower limb spasticity, initiating treatment with BoNT earlier after stroke (≤24 months vs. >24 months) was associated with significant benefits in a greater number of efficacy outcomes than later treatment initiation [32]. In contrast, in the current study, response rates for all three investigated DAS domains were either consistent across the assessed time periods since stroke or appeared to be higher with increased time since stroke, and the difference in treatment effect between incobotulinumtoxinA and placebo was actually largest in those with >10 years since stroke when upper limb position abnormality and dressing disability were considered. This demonstrates that incobotulinumtoxinA efficacy can still be achieved in patients who have gone untreated for spasticity for many years, and treatment with incobotulinumtoxinA can be started in patients with spasticity at any time, regardless of when their spasticity was first diagnosed. Similarly, improvements were significantly better in patients who received one or two cycles of abobotulinumtoxinA “late” in the disease course than those who received it “early” poststroke, although benefits in achieving an individual functional goal, and ease of care and hygiene, were seen irrespective of timing of treatment with respect to stroke, particularly after four injection cycles [33].

Repeated injections were administered in five of the six pooled studies. Multiple injection cycles of incobotulinumtoxinA have been shown to have a cumulative effect on DAS pain in spasticity [29] and pain in cervical dystonia [34]. Our analyses of the remaining three DAS domains showed that many patients had a maintained response to incobotulinumtoxinA at the time of reinjection. In common with other BoNT-A analyses [21,35], we observed that the effects of repeated injection cycles of incobotulinumtoxinA at 4 weeks postinjection appeared to plateau after about the third cycle. However, the beneficial effects on DAS scores (measured using the PTT) of incobotulinumtoxinA at 12 weeks postinjection were not found to have plateaued after four injection cycles in a post hoc analysis of data from two trials [36].

Many study publications using the DAS tool focus on only the PTT, either pooling findings across the PTT domains [14,17,19,37,38] or reporting domain-specific data only from patients for whom the domain was the PTT [13,18], including a meta-analysis of five BoNT-A studies [39]. There is some evidence showing that BoNT-A improves each of the four domains [12,13,35], although significant improvement may not be possible for domains that are only mildly impaired at baseline [40,41].

Spasticity develops in about 25% of stroke survivors, although the incidence varies according to the follow-up time poststroke since spasticity can develop late or resolve; for example, in a meta-analysis of data from 24 studies, the incidence was 31.6% if follow-up was within 1 month, 21.8% if in 1–3 months, 26.3% if in 3–6 months, and 24.2% if after more than 6 months [7]. The findings of this analysis show that, overall, efficacy was not affected by the timing of incobotulinumtoxinA relative to stroke, which will, therefore, be of interest to many patients who have survived stroke.

The strengths of this study include that the patient population came from a wide (international) geographic spread and that the DAS, a standard measure of functional disability, was used across the six studies included in the analyses. Additional strengths include that most of the incorporated studies were placebo-controlled for the first injection cycle, data were obtained over multiple incobotulinumtoxinA injection cycles and the analyses were performed on a background of stable antispastic medications (centrally acting muscle relaxants, benzodiazepine) and physical/occupational therapy. Using a multipattern treatment approach, incobotulinumtoxinA produced effective improvements in QoL-related outcomes, a finding that should be considered along with the well-established safety profile and low immunogenicity profile of incobotulinumtoxinA [42,43,44,45,46,47,48,49,50,51,52,53,54,55,56,57,58,59,60,61,62,63,64,65,66,67,68,69,70,71,72,73,74,75,76,77,78]. Furthermore, the mixed etiology of spasticity evident in this study may make the findings applicable to a wider range of patients, although most patients had poststroke spasticity.

Limitations of this study include potential bias due to different patient numbers from the different studies used in the pooled analyses. For example, the PURE study [14] contributed around 40% of patients analyzed, whereas study MRZ_60201_03071 contributed only 3%. Additionally, the contributing studies were not all in the same drug development phase. Their designs differed, including two that were not placebo-controlled, and the DAS was not defined as a primary endpoint in most of the studies. Finally, as with other published pooled analyses, we have not used a systematic search approach and instead only selected relevant clinical trials conducted by the study sponsor - Merz Therapeutics GmbH. The reason for this was to ensure data availability at the level required to conduct the pooled analyses.

## 4. Conclusions

This pooled analysis showed that incobotulinumtoxinA significantly improves QoL-related outcomes, such as upper limb positioning abnormality and dressing- and hygiene-related disability, in adults with upper limb spasticity. This is the largest patient cohort analyzed to date in this setting, providing additional evidence to support the use of incobotulinumtoxinA in QoL-related outcomes. The three DAS domains analyzed are frequently selected by patients with spasticity as the PTT, indicating the impact they have on patients’ lives.

The effects of incobotulinumtoxinA 4 weeks after injection were maintained or enhanced over multiple injection cycles for all three DAS domains, supporting the use of repeated injection cycles to provide sustained QoL benefit. IncobotulinumtoxinA represents an important treatment option to achieve better QoL-related outcomes for patients with upper limb spasticity, irrespective of the duration of their condition. Improvements in QoL induced by incobotulinumtoxinA can be achieved regardless of the underlying neurological condition that has resulted in spasticity, sex, ethnicity, and pretreatment status. Although it is desirable to start treating spasticity early, the results of this analysis demonstrate that initiating incobotulinumtoxinA treatment later will still lead to meaningful QoL improvements for the patients. Future studies on the benefit of BoNT therapy for QoL are warranted to further understand these improvements in patients with spasticity.

## 5. Materials and Methods

### 5.1. Studies Included in the Analysis

This post hoc analysis used pooled upper limb position abnormality, dressing and hygiene data from six prospective, multicenter, phase 2 or 3 studies of incobotulinumtoxinA (Xeomin^®^; Merz Therapeutics GmbH, Frankfurt, Germany) in the treatment of upper [12,14,17,18,30] or upper and lower [15] limb muscle spasticity in adults conducted by the study sponsor - Merz Therapeutics GmbH (Table 3). Studies were limited to Merz-sponsored studies in order to ensure sufficient data availability to conduct the pooled analyses.

MRZ_60201_03071 was terminated prematurely due to low recruitment. It was not registered and no data have been published previously outside of pooled analyses. Four of the studies were randomized, double-blind, and placebo-controlled [12,14,17,30], whereas the remaining two studies evaluated different incobotulinumtoxinA dosing schedules [15] and dilutions [18].

The studies were conducted across the world and included a range of patient ethnicities. In the six studies, adult patients aged ≥18 years who had not received BoNT-A injections within at least 4 months of screening received incobotulinumtoxinA injections as appropriate for their condition, most commonly at a total body dose of 400 U. Each injection was followed by at least 12 weeks of observation and assessment. In studies where patients received more than one incobotulinumtoxinA injection, the time between injections was 12–14 weeks for the majority of patients [29].

All studies were conducted in accordance with the Declaration of Helsinki and Good Clinical Practice and were approved by the ethics committee for each participating site. All patients provided written informed consent prior to study participation.

### 5.2. Disability Assessment Scale

The current analyses used the limb position, dressing, and hygiene domains of the DAS [20] to measure functional disability and activity limitations resulting from spasticity in each of the three domains and, therefore, provide further information regarding the impact of upper limb spasticity on QoL. In all studies, these domains were assessed at baseline, every injection visit, and 4 weeks after each injection using a 4-point rating scale: score 0 (no disability), 1 (mild disability), 2 (moderate disability), and 3 (severe disability).

Response was defined as ≥1-point reduction from baseline in limb position abnormality, dressing, and hygiene score, as per previous studies [35,37,79]. Apart from study MRZ_60201_03071, where no PTT was selected, the PTT was selected by the patient and physician from one of the four DAS items (limb position, dressing, hygiene, pain) with a required baseline score ≥2.

### 5.3. Analyses

Separate analyses for each DAS domain were performed using pooled data from patients with each limb position abnormality, dressing-, and hygiene-related disability at baseline (DAS score ≥1 for that domain). Patients with more than one domain score ≥ 1 could be included in multiple analyses.

DAS response rates over time for all injection cycles relative to baseline were recorded at injection visit 1 (baseline), at each subsequent injection visit if applicable, and at control visits 4 weeks (±3 days) after each injection. Response rates for incobotulinumtoxinA versus placebo were compared 4 weeks after the first injection using Wald tests (95% CI) and evaluated 4 weeks after each injection visit of the subsequent three cycles for incobotulinumtoxinA without placebo control. For the placebo-controlled studies, after the placebo injection cycle (first cycle only), data from patients who subsequently received incobotulinumtoxinA in an open-label extension phase were included with data from the original incobotulinumtoxinA groups (data assigned to first, second, and third incobotulinumtoxinA doses).

Data for limb position abnormality, dressing-, and hygiene-related disability at week 4 following incobotulinumtoxinA injection were analyzed in terms of the proportion of incobotulinumtoxinA- and placebo-treated patients who responded to treatment and the likelihood of response to incobotulinumtoxinA versus placebo (logistic regression analysis, presented as an OR analyzed using the chi-square test and 95% Wald CI). Furthermore, several subgroup analyses were performed using data from the first injection cycle. The difference in overall response rates to incobotulinumtoxinA versus placebo according to baseline severity of the respective DAS domain (mild, moderate, severe) was assessed, as well as the difference in overall response rates to incobotulinumtoxinA versus placebo according to time since stroke (0–2 years, 3–5 years, 6–10 years, >10 years). The proportion of incobotulinumtoxinA- and placebo-treated patients who responded to treatment according to PTT (any of the four DAS domains) was also evaluated after all injection cycles.

Analyses were based on observed cases; there was no strategy for missing postbaseline data in participants with a domain DAS score ≥1 at baseline, as few data were missing (limb position abnormality, 2.5% (*n* = 23), dressing disability, 2.4% (*n* = 22), hygiene-related disability, 2.4% (*n* = 21)). Analyses were performed using Statistical Analysis Software (SAS) 9.4 (SAS Institute Inc., Cary, NC, USA). Logistic regression was used to calculate ORs and associated *p*-values. For response differences, Wald tests and 95% CIs were computed.

## Figures and Tables

**Table 1:** Characteristics of patients (*n* = 937) with limb position abnormality and dressing- and hygiene-related disability (score ≥1) at baseline.

DAS Domain	Limb Position			Dressing			Hygiene		
Characteristic	INCO (*n* = 699)	Placebo (*n* = 219)	Total (*n* = 918)	INCO (*n* = 690)	Placebo (*n* = 217)	Total (*n* = 907)	INCO (*n* = 655)	Placebo (*n* = 210)	Total (*n* = 865)
Mean ± SD age, years	55.9 ± 12.8	56.3 ± 12.2	56.0 ± 12.6	56.1 ± 12.6	56.2 ± 1 2.3	56.1 ± 12.5	56.2 ± 12.6	56.4 ± 12.3	56.2 ± 12.5
Sex, n (%)									
Male	435 (62.2)	136 (62.1)	571 (62.2)	430 (62.3)	134 (61.8)	564 (62.2)	403 (61.5)	128 (61.0)	531 (61.4)
Female	264 (37.8)	83 (37.9)	347 (37.8)	260 (37.7)	83 (38.2)	343 (37.8)	252 (38.5)	82 (39.0)	334 (38.6)
Ethnicity, n (%)									
White	535 (76.5)	170 (77.6)	705 (76.8)	530 (76.8)	169 (77.9)	699 (77.1)	506 (77.3)	166 (79.1)	672 (77.7)
Black or African American	11 (1.6)	3 (1.4)	14 (1.5)	10 (1.5)	3 (1.4)	13 (1.4)	10 (1.5)	3 (1.4)	13 (1.5)
Asian	95 (13.6)	46 (21.0)	141 (15.4)	95 (13.8)	45 (20.7)	140 (15.4)	89 (13.6)	41 (19.5)	130 (15.0)
Other	6 (0.9)	0	6 (0.7)	6 (0.9)	0	6 (0.7)	5 (0.8)	0	5 (0.6)
Missing	52 (7.4)	0	52 (5.7)	49 (7.1)	0	49 (5.4)	45 (6.9)	0	45 (5.2)
Mean ± SD height, cm	168.3 ± 9.5 ^a^	168.9 ± 8.2	168.5 ± 9.2	168.3 ± 9.5 ^b^	168.7 ± 8.2	168.4 ± 9.2	168.2 ± 9.5 ^c^	168.6 ± 8.3	168.3 ± 9.2
Mean ± SD weight, kg	75.4 ± 14.9 ^d^	75.3 ± 14.5	75.3 ± 14.8	75.4 ± 14.9 ^e^	75.2 ± 14.6	75.4 ± 14.8	75.5 ± 14.8 ^f^	75.4 ± 14.7	75.5 ± 14.8
BoNT-A naïve, n (%)	324 (46.4)	154 (70.3)	478 (52.1)	321 (46.5)	155 (71.4)	476 (52.5)	310 (47.3)	150 (71.4)	460 (53.2)
Etiology of spasticity, n (%)									
Stroke	652 (93.3)	218 (99.5)	870 (94.8)	648 (93.9)	216 (99.5)	864 (95.3)	616 (94.1)	210 (100.0)	826 (95.5)
Multiple sclerosis	1 (0.1)	1 (0.5)	2 (0.2)	1 (0.1)	1 (0.5)	2 (0.2)	1 (0.2)	0	1 (0.1)
Infantile cerebral palsy	6 (0.9)	0	6 (0.7)	6 (0.9)	0	6 (0.7)	3 (0.5)	0	3 (0.4)
Brain injury	22 (3.2)	0	22 (2.4)	20 (2.9)	0	20 (2.2)	19 (2.9)	0	19 (2.2)
Other	18 (2.6)	0	18 (2.0)	15 (2.2)	0	15 (1.7)	16 (2.4)	0	16 (1.9)
DAS score ^g^ at baseline, n (%)									
1 = mild	74 (10.6)	22 (10.0)	96 (10.5)	114 (16.5)	27 (12.4)	141 (15.6)	140 (21.4)	25 (11.9)	165 (19.1)
2 = moderate	345 (49.4)	123 (56.2)	468 (51.0)	380 (55.1)	128 (59.0)	508 (56.0)	340 (51.9)	122 (58.1)	462 (53.4)
3 = severe	280 (40.1)	74 (33.8)	354 (38.6)	196 (28.4)	62 (28.6)	258 (28.5)	175 (26.7)	63 (30.0)	238 (27.5)
Mean ± SD time since diagnosis of spasticity, years	5.5 ± 6.5	3.9 ± 5.0	5.1 ± 6.2	5.5 ± 6.4	3.9 ± 5.0	5.1 ± 6.1	5.3 ± 6.2	4.0 ± 5.0	5.0 ± 6.0

Missing height data: ^a^ one, ^b^ one, and ^c^ one patient(s). Missing weight data: ^d^ one, ^e^ three, and ^f^ three patient(s).^g^ DAS score for limb position abnormality, dressing, and hygiene. BoNT-A, botulinum toxin type A; DAS, Disability Assessment Scale; INCO, incobotulinumtoxinA; SD, standard deviation.

**Table 2:** DAS domain response rates (≥1-point improvement in DAS score) at week 4 after the first injection cycle by treatment and time since stroke.

Time Since Stroke (Years)	IncobotulinumtoxinA			Placebo			Difference (IncobotulinumtoxinA–Placebo)		
	Participants with Data (n)	Response Rate (%)	95% CI	Participants with Data (n)	Response Rate (%)	95% CI	Response Rate (%)	95% CI	*p*-Value
Limb position abnormality									
0–2	262	44.3	[38.3–50.3]	115	23.5	[15.7–31.2]	20.8	[11.0–30.6]	<0.0001
3–5	169	45.0	[37.5–52.5]	54	16.7	[6.7–26.6]	28.3	[15.9–40.8]	<0.0001
6–10	143	45.5	[37.3–53.6]	31	12.9	[1.1–24.7]	32.6	[18.2–46.9]	<0.0001
>10	94	53.2	[43.1–63.3]	17	11.8	[0.0–27.1]	41.4	[23.1–59.8]	<0.0001
Dressing disability									
0–2	261	34.5	[28.7–40.2]	113	21.2	[13.7–28.8]	13.2	[3.8–22.7]	0.0063
3–5	167	26.9	[20.2–33.7]	54	13.0	[4.0–21.9]	14.0	[2.8–25.2]	0.0144
6–10	141	29.8	[22.2–37.3]	31	19.4	[5.4–33.3]	10.4	[−5.4–26.3]	0.1963
>10	91	36.3	[26.4–46.1]	17	17.6	[0.0–35.8]	18.6	[−2.0–39.3]	0.0771
Hygiene-related disability									
0–2	251	31.1	[25.4–36.8]	106	17.0	[9.8–24.1]	14.1	[4.9–23.3]	0.0026
3–5	159	32.1	[24.8–39.3]	54	20.4	[9.6–31.1]	11.7	[−1.3–24.7]	0.0768
6–10	134	38.1	[29.8–46.3]	31	22.6	[7.9–37.3]	15.5	[−1.4–32.3]	0.0719
>10	83	32.5	[22.5–42.6]	17	23.5	[3.4–43.7]	9.0	[−13.5–31.5]	0.4339

Wald test was used to determine between-treatment *p*-values. CI, Wald confidence interval; DAS, Disability Assessment Scale.

**Table 3:** The six studies included in the pooled analyses (main period and open-label extension period).

Study Name/NCT Number (Merz ID) (Reference)	Phase	Countries	Study Design and Objective	Study Period	Primary Outcome	Treatment (Total Body Dose)	Subjects and Indication
(MRZ_60201_03071) [30] Not published (study terminated due to low recruitment)	2	Germany	Prospective, randomized, double-blind, placebo-controlled, parallel-group, multicenter pilot study (12 weeks) to investigate the efficacy and safety of incobotulinumtoxinA in the treatment of pain in upper limb spasticity	Main period	Mean evening pain intensity measured using the 11-point Box Scale	One treatment cycle: IncobotulinumtoxinA (up to 400 U; range: 240–400 U) Placebo	*n* = 14 adults with pain caused by upper limb spasticity due to multiple etiologies
NCT00432666 (MRZ_60201_0410) (Kanovsky et al. [12]; Kanovsky et al. [13])	3	Czech Republic, Hungary, Poland	Prospective, randomized, double-blind, placebo-controlled, parallel-group, multicenter trial (20 weeks) with an open-label extension period (69 weeks) to investigate the efficacy and safety of incobotulinumtoxinA in the treatment of poststroke upper limb spasticity	Main Period	Wrist flexor response rate (≥1-point improvement in AS score) at week 4	One treatment cycle incobotulinumtoxinA (intended up to 400 U; median 320 U; range: 80–435 U) Placebo	*n* = 148 adults with poststroke upper limb spasticity
				OLEX		Five treatment cycles: IncobotulinumtoxinA (intended up to 400 U; 1st cycle median 385 U, others 400 U)	*n* = 145 adults (from main period)
NT-SPIN NCT00465738 (MRZ_60201_06071) (Barnes et al. [18])	3	Austria, France, Germany, Italy, Portugal, Spain, Switzerland, United Kingdom	Prospective, randomized, observer-blind, parallel-group, multicenter trial (20 weeks) to assess efficacy and safety of two different dilutions of incobotulinumtoxinA in patients with upper limb spasticity	Main Period	DAS response rate (≥1-point improvement) at week 4 ^a^	One treatment cycle: IncobotulinumtoxinA (two dilutions: (20 or 50 U/mL) (intended up to 400 U; median 300 U; actual up to 495 U)	*n* = 192 adults with stable upper limb spasticity of diverse etiology
PURE NCT01392300 (MRZ_60201_SP3001) (Elovic et al. [14]; Marciniak et al. [79]; Marciniak et al. [16])	3	Czech Republic, Germany, Hungary, India, Poland, Russian Federation, United States of America	Prospective, randomized, double-blind, placebo-controlled, parallel-group, multicenter study (12 weeks) with an open-label extension period (36 weeks) to investigate the efficacy and safety of incobotulinumtoxinA in the treatment of poststroke upper limb spasticity	Main Period	Change in muscle tone from baseline to week 4, measured using the AS ^b^	One treatment cycle: IncobotulinumtoxinA (400 U) Placebo	*n* = 317 adults with poststroke upper limb spasticity
				OLEX		Three treatment cycles: IncobotulinumtoxinA (400 U)	*n* = 299 (from main period)
TOWER NCT01603459 (MRZ_60201_3053) (Wissel et al. [15])	3	Canada, France, Germany, Italy, Norway, Portugal, Spain, United States of America	Prospective, nonrandomized, open-label, single-arm, multicenter dose-titration study (48 weeks) to investigate the safety and efficacy of incobotulinumtoxinA in subjects requiring doses of 800 U during the course of the study for the treatment of upper and lower limb spasticity	Main Period	Safety	Three treatment cycles: IncobotulinumtoxinA (IC1: 400, IC2: 600, IC3: ≤800 U ^c^),	*n* = 155 adults with chronic upper and lower limb spasticity of the same body side due to cerebral causes
J-PURE JapicCTI Number: CTI-153029 (MRZ_60201_30991) (Masakado et al. [17])	3	Japan	Prospective, randomized, double-blind, placebo-controlled, parallel-group, multicenter study (52 weeks in total), with an open-label lead-in tolerability period (1 week), a main study period (12 weeks) and an open-label extension period (32–40 weeks), to investigate the efficacy and safety of two different doses of incobotulinumtoxinA in the treatment of poststroke upper limb spasticity	Main Period	Change in muscle tone from baseline to week 4, measured using the modified AS	One treatment cycle: IncobotulinumtoxinA (400 U or 250 U) Placebo	*n* = 100 adults with poststroke upper limb spasticity
				OLEX		Three treatment cycles: IncobotulinumtoxinA (400 U)	*n* = 90 (from main period)

^a^ In this study, patients chose one of four domains of the DAS (dressing, limb position, pain, hygiene) as the primary therapeutic target; this was limb position in 63%, dressing in 24%, pain in 6%, and hygiene in 8% of patients. ^b^ In PURE, the primary target clinical pattern treated included flexed elbow, flexed wrist, or clenched fist at predefined fixed doses. Other clinical patterns could be treated with the remainder of the total dose as medically indicated. ^c^ Scheduled doses were administered to 91.0% of patients in IC1, 90.8% in IC2, and 82.9% in IC3 (93.6% of patients received a dose of ≥700 U in IC3). AS, Ashworth Scale; DAS, Disability Assessment Scale; IC, injection cycle; LITP, lead-in tolerability period; MP, main period; OLEX, Open-Label Extension.

## Data Availability

Primary data from five of the six pooled studies are published in the respective peer-reviewed manuscripts cited in the text. Data from the sixth study (MRZ_60201_03071) are not publicly available as study unpublished (terminated due to low recruitment).

## Supplementary Materials

The following supporting information can be downloaded at: https://www.mdpi.com/article/10.3390/toxins16010019/s1, Table S1: Characteristics of patients with principle therapeutic target-related disability (any DAS domain) at baseline; Figure S1: Change in Disability Assessment Scale domain scores at week 4 after the first injection cycle by treatment.

## Abbreviations

AS: Ashworth Scale; BoNT-A: botulinum toxin type A; CI: confidence interval; CV: control visit; DAS: Disability Assessment Scale; HRQoL: health-related QoL; IC: injection cycle; INCO, incobotulinumtoxinA; IV: injection visit; LITP: lead-in tolerability period; MP: main period; OLEX: Open Label Extension; PTT: principal therapeutic target; PRISM: Patient Reported Impact of Spasticity Measure; OR: odds ratio; QoL: quality of life; SD, standard deviation.
