# Supplementary material for: Improvement in Quality-of-Life-Related Outcomes Following Treatment with IncobotulinumtoxinA in Adults with Limb Spasticity: A Pooled Analysis
Source: Toxins (Basel). 2023 Dec 29;16(1):19. doi: 10.3390/toxins16010019 (PMC10821091; doi:10.3390/toxins16010019)

Supplementary Materials: Improvement in quality-of-life-related outcomes following treatment with incobotulinumtoxinA in adults with limb spasticity: a pooled analysis

Table S1. Characteristics of patients with principle therapeutic target-related disability (any DAS domain) at baseline.

Characteristic	INCO (N=684)	Placebo (N=215)	Total (N=899)
**Mean ± SD age, years**	55.7 ± 12.8	56.1 ± 12.3	55.8 ± 12.7
**Male, n (%)**	422 (61.7)	134 (62.3)	556 (61.9)
**Ethnicity, n (%)**			
White	519 (75.9)	166 (77.2)	685 (76.2)
Black or African American	11 (1.6)	3.0 (1.4)	14 (1.6)
Asian	96 (14.0)	46 (21.4)	142 (15.8)
Other	6 (0.9)	0	6 (0.7)
Missing	52 (7.6)	0	52 (5.8)
**Mean ± SD height, cm**	168.3 ± 9.5[a]	168.7 ± 8.2	168.4 ± 9.2
**Mean ± SD weight, kg**	75.3 ± 14.9[b]	75.5 ± 14.5	75.3 ± 14.8
**BoNT-A naïve, n (%)**	319 (46.6)	154 (71.6)	473 (52.6)
**Aetiology of spasticity, n (%)**			
Stroke	639 (93.4)	215 (100.0)	854 (95.0)
Multiple sclerosis	1 (0.2)	0	1 (0.1)
Infantile cerebral palsy	5 (0.7)	0	5 (0.6)
Brain injury	21 (3.1)	0	21 (2.3)
Other	18 (2.6)	0	18 (2.0)
**DAS score at baseline, n (%)**			
1 = mild	3 (0.4)	0	3 (0.3)
2 = moderate	344 (50.3)	123 (57.2)	467 (52.0)
3 = severe	337 (49.3)	92 (42.8)	429 (47.7)

**Mean ± SD time since diagnosis of spasticity, years**	5.5 ± 6.3	3.9 ± 5.0	5.1 ± 6.0

[a]Missing height data: one patient.
[b]Missing weight data: three patient(s).

BoNT-A, botulinum toxin type A; DAS, Disability Assessment Scale; INCO, incobotulinumtoxinA; SD, standard deviation.

Figure S1: Change in Disability Assessment Scale domain scores at week 4 after the first injection cycle by treatment.

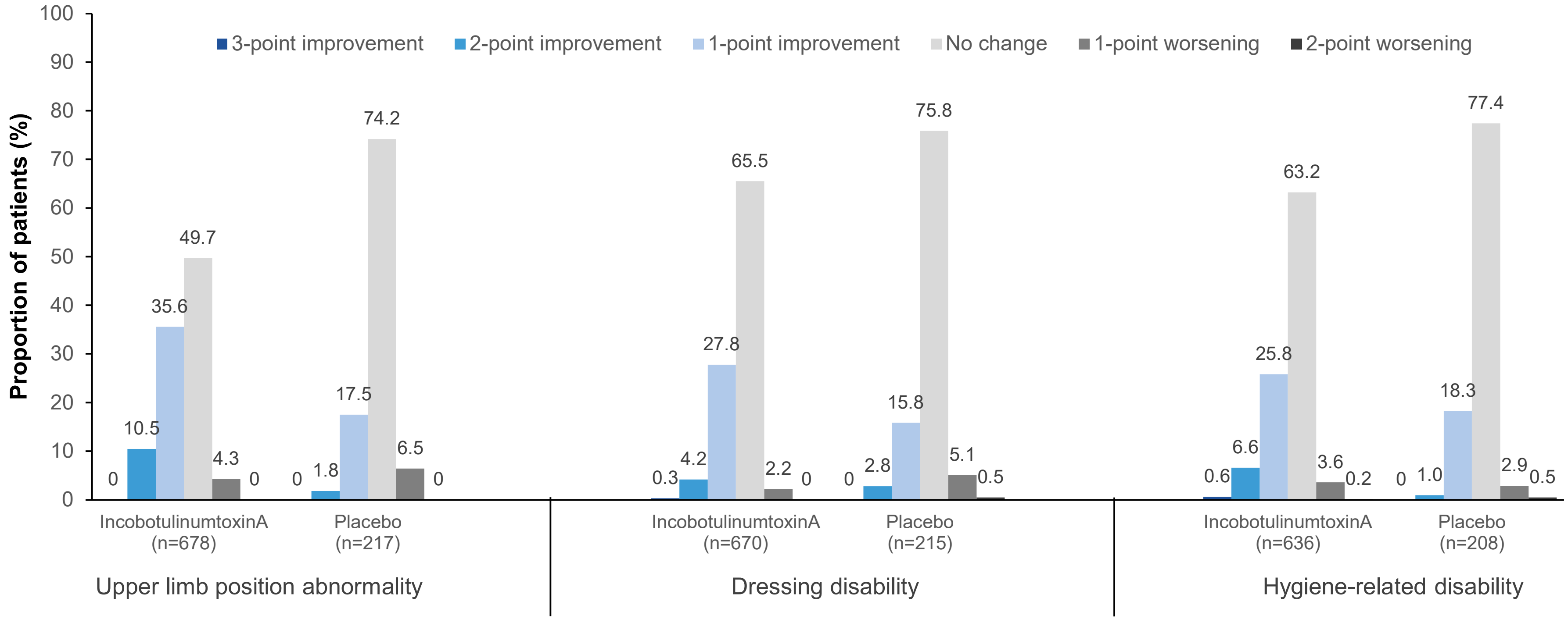

Supplement: Supplementary file 1 [file toxins-16-00019-s001.zip › toxins-2683283-supplementary.pdf]
